# The influence of psychological factors on the outcomes of laparoscopic Nissen fundoplication

**DOI:** 10.1186/1750-1164-1-2

**Published:** 2007-02-20

**Authors:** Laurent Biertho, Dutta Sanjeev, Herawati Sebajang, Marty Antony, Mehran Anvari

**Affiliations:** 1 Centre for Minimal Access Surgery, St. Joseph's Healthcare, McMaster University, Hamilton, ON, Canada; 2 Department of Psychology, St. Joseph's Healthcare, McMaster University, Hamilton, ON, Canada

## Abstract

**Background:**

Psychological factors play a role in a variety of gastrointestinal illness, including gastroesophageal reflux disease (GERD). Their impact on the surgical outcomes of antireflux surgery is unknown.

**Methods:**

This is a single institution prospective controlled trial, comparing patients undergoing a laparoscopic Nissen fundoplication for GERD (LNF Group, n = 17) to patients undergoing an elective laparoscopic cholecystectomy for biliary colic (Control Group, n = 10). All patients had a psychological assessment before surgery, at 3 months and 6 months after surgery (i.e. Symptom CheckList-90-R somatization subset (SCL-90-R), Depression Anxiety Stress Scales, Anxiety sensitivity index, Illness attitude scale and Beck Depression Inventory II). GERD symptoms were recorded in the LNF Group using a standardized questionnaire (score 0–60). Patients with post-operative GERD symptoms score above 12 at 6 months were evaluated specifically. Statistical analysis was performed using a Student T test, and statistical significance was set at 0.05.

**Results:**

There was no significant difference in preoperative and postoperative psychological assessment between the two groups. In the LNF Group, 7 patients had persisting GERD symptoms at 6 months (GERD score greater than 12). The preoperative SCL-90-R score was also significantly higher in this subgroup, when compared to the rest of the LNF Group (18.2 versus 8.3, p < 0.05) and to the Control Group (18.2 versus 7.9, p < 0.05). There was no significant difference for the other psychological tests.

**Conclusion:**

The SCL-90-R Somatization Subset, reflecting the level of somatization in a patient, may be useful to predict poor outcomes after antireflux surgery. Cognisance of psychological disorders could improve the selection of an optimal treatment for GERD and help reduce the rate of ongoing symptoms after LNF.

## Background

Laparoscopic approach had a significant impact in the modern treatment of most abdominal pathologies, including gastroesophageal reflux disease (GERD). Multiple well-designed trials have confirmed that laparoscopic Nissen fundoplication (LNF) compares favorably with its open counterpart [1-3].

However, a small but significant number of patients (12% at 5 years in our initial experience) have persisting or recurrent GERD symptoms after antireflux surgery [4,5]. When a surgical complication has been ruled out, poor patient selection, psychological disorders associated or mimicking GERD (i.e. somatization, anxiety or depressive disorders), can play a role. Pre-operative identification of such psychological disorders could help orient patients to a psychological therapy instead of surgery.

The aim of this study is to identify psychological factors associated with poor surgical outcomes after antireflux surgery.

## Materials and methods

This is a single institution prospective controlled trial, comparing patients undergoing a laparoscopic Nissen fundoplication for GERD (LNF Group, n = 17, 13 females and 4 males) to patients undergoing an elective laparoscopic cholecystectomy for biliary colic (Control Group, n = 10, 9 females and 1 male). Patients were prospectively recruited between March 2002 and May 2003, from the outpatient consultations of a University Medical Centre (Centre for Minimal Access Surgery, McMaster University, Hamilton, Ontario).

The LNF Group was recruited among patients who were considering surgical treatment for the management of classical symptoms of GERD. All patients had pre-operative 24-hour pH monitoring, with GERD defined as a percentage time with pH<4 greater than 4% during 24 h, and a Demeester score greater that 14.3. Preoperative workup also included esophageal manometry and esophago-gastro-duodenoscopy.

A detailed questionnaire was used to assess GERD specific symptoms (heartburn, pain, regurgitation, dysphagia and fullness) and non-GERD symptoms (vomiting, constipation, diarrhea, loss of appetite and cough). Each symptom was scored as a product of severity (0 = none, 3 = severe) and frequency (0 = none, 4 = daily), for a GERD symptoms score ranging between 0 and 72 and a non-GERD symptoms score ranging between 0 and 48. This symptom score has been validated previously [6].

Patients with post-operative GERD symptoms score above 12 at 6 months (LNF Poor outcomes Group) were compared to the rest of the LNF Group (LNF Good outcomes Group). A score of 12 was chosen based on our previous experience with the GERD symptoms score (SS)[6]. The SS showed a high responsiveness index, after LNF, which indicate that the instrument is highly sensitive. The minimal clinically important difference is about seven scale points. A 12 points score (on a 0 to 72 points scale) was considered to be a good cut-off point to consider that patients have ongoing or recurrent GERD symptoms.

The Control Group consisted in patients with typical biliary pain with radiologic evidence of cholelithiasis. This group represents patients with a specific disease entity that is expected to be cured with a surgical treatment.

Patients with known psychiatric or psychologic disorders were excluded, as well as patients with chronic pain syndrome.

The two groups had the same psychological assessment one month before surgery, at 3 months and 6 months after surgery. The following tests were administered:

- Symptom CheckList-90-R, somatization subset (SCL-90-R): a 12-item questionnaire that measures a current point-in-time somatization symptom complex as outlined in the DSM IV-R [7]. Each item is scored on a 5-point scale (0 to 4). A raw score can be calculated by dividing the summed total score by the number of items. That score is then referred to gender-appropriate norms for conversion to standard t-score, which may alternatively be expressed as a percentile. T-scores equal to or greater than 63 (90^th ^percentile) for the global score index (on the 90 items) or in any two symptom dimensions warrants clinical workup. In this study, the summed total score was used for evaluation of somatization level.

- Depression Anxiety Stress Scales (DASS): a 21-item instrument for distinguishing between features of depression, physical arousal, and psychological tension and agitation in clinical and non-clinical groups. It has acceptable to excellent internal consistency and concurrent validity [8]. Clinical range, based on normative data, is defined as scores of 16.57 or above for stress, 12.75 or above for anxiety, and 9.26 or above for depression.

- Anxiety Sensitivity Index (ASI): a 16-item self-report questionnaire measuring fear of anxiety-related symptoms. Each item is scored using a five point Likert type scale with endpoints of "very little" (0 points) and "very much" (4 points). The ASI yields a total score obtained by summing the rating across all items, with higher scores reflecting higher level of fear and concern regarding physical symptoms of anxiety. Psychometric data is available for community and clinical samples [9];

- Illness Attitude Scale (IAS): a 29-item questionnaire that measures attitudes, fears and beliefs associated with the psychopathology of hypochondriasis and that of abnormal illness behavior. All items are self-rated on 5 points Likert scales, with endpoints of "no" (zero points) and "most of the times" (four points). The scores on the two subscales (Health Anxiety and Illness Behaviour) appear to differentiate between general medical outpatients (mean of 10.9 and 9.0 respectively), general practice patients (11.3 and 6.8) and subjects from the general population (9.1 and 4.7) [10,11].

- Beck Depression Inventory II (BDI): a 21-item scale designed to measure clinical depression, shown to have excellent psychometric properties. The original authors recommended cut-off scores for non depressed (<10), mildly depressed (10–14), moderately depressed (15–22) and severely depressed (23 +) [12].

Ethic Board approval was obtained to collect these data.

A similar technique of laparoscopic cholecystectomy or laparoscopic Nissen fundoplication was used in all patients. In short, the surgical technique of LNF consisted in a 2 to 3 cm, 360 degree wrap with selective division of short gastric vessels and crura repair [5]. The technique of laparoscopic cholecystectomy included the dissection of Callot's triangle, identification and control of the cystic duct and cystic artery and retrograde dissection of the gallbladder bed.

Data were entered in an electronic database and results are expressed as a Mean ± Standard Deviation. Statistical analysis was performed using a Student T tests and statistical significance was set at 0.05.

## Results

The results of the pre-operative and 6-months psychological tests are summarized in Table 1. There was no significant difference between the LNF Group and the Control group. Five patients in the LNF group had abnormal Beck depression inventory's scores that could suggest depressive disorders (mild (2), moderate (2) and severe (1). Three patients normalized their BDI score 6 months after surgery, one patient went from a score of 19 (severe) to 10 (mild) and the last one had an increase from 10 (mild) to 17 (moderate).

**Table 1:** Psychological scores before surgery and at 6 months (Mean ± Standard Deviation).

	LNF Group		Control Group	
	Pre-op	6 months	Pre-op	6 months

SCL-90	12 ± 9	5.5 ± 4.6	7.9 ± 4.6	7.2 ± 5.5
DASS	10.7 ± 8.8	5.3 ± 8.8	5.6 ± 4	10.8 ± 13
Anxiety SI	12.2 ± 8.8	8.8 ± 10.2	8.6 ± 6	10.6 ± 11
Illness AS	30.8 ± 11	24.3 ± 9.9	33.4 ± 10	30.3 ± 16
BDI	8 ± 7	3.7 ± 4.5	6.4 ± 5.2	8.7 ± 11.9

There was also a significant decrease in GERD symptoms score in the LNF Group (39.8 ± 13.2 preop versus 15.7 ± 17 at 6 months, p < 0.05), but also a significant decrease in the non-GERD symptoms score (11.8 ± 10.6 preop versus 4.3 ± 8.4 at 6 months, p < 0.05). This was correlated with a significant drop in the percentage time below pH4 during 24 h and DeMeester score (p < 0.05) (Table 2).

**Table 2:** Evolution in 24 h pH monitoring (Mean ± Standard Deviation).

	24 h pH monitoring		DeMeester score	
	Pre-op	6 months	Pre-Op	6 months

Whole group	7.4 ± 4.6	1.4 ± 2.5	30.4 ± 19.1	6.3 ± 12.1
LNF Poor outcomes	7.3 ± 9.6	0.8 ± 0.8	34.3 ± 40	3 ± 2.8
LNF Good outcomes	7.5 ± 3.3	1.7 ± 2.4	28.7 ± 12.8	7.9 ± 11.8

In the LNF Group, 7 patients had a GERD symptoms score greater than 12 at 6 months (on a 0 to 72 scale) and constitute the LNF-Poor outcomes Group. This group was compared to the other 10 patients in the LNF Group (LNF-Good outcomes Group). Changes in 24 h pH results and DeMeester scores are summarized in Table 2. None of the patients with ongoing GERD symptoms had objective acid reflux, as demonstrated by normal 24 h pH monitoring.

**Figure 1:**
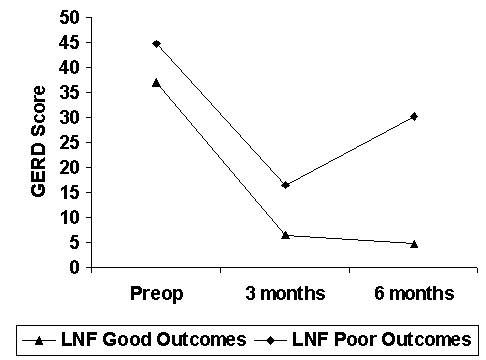
GERD Score, Good versus Poor outcomes Groups (Mean).

**Figure 2:**
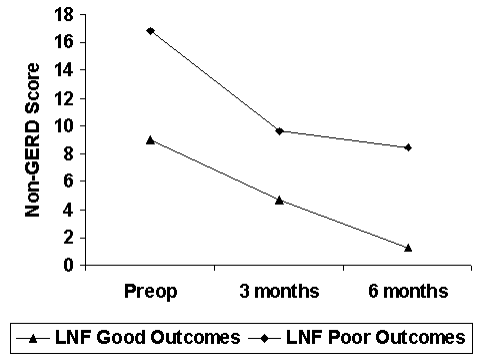
Non-GERD Score, Good versus Poor Outcomes Groups (Mean).

The evolution of GERD and non-GERD symptoms score in each group is summarized in Figure 1 and 2 respectively. The difference in non-GERD symptoms scores was not significant.

**Figure 3:**
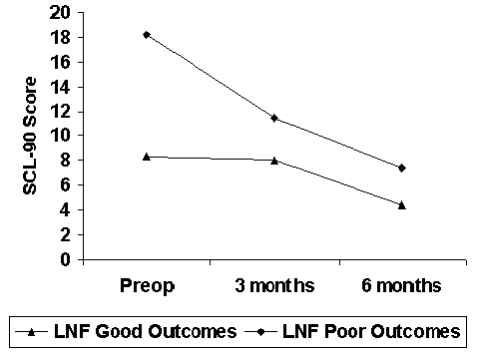
SCL-90 Score, Good versus Poor Outcomes Groups (Mean).

The results of psychological tests were also compared between the two groups. A significantly higher preoperative SCL-90-R score was found in patients with ongoing symptoms (18.2 versus 8.3, p < 0.05, Figure 3), reflecting a higher level of somatization in these patients. There was no significant difference in the other psychological tests.

## Discussion

Psychological disorders can play a role in a variety of gastrointestinal illness, including esophageal diseases. The influence of psychological factors in gastroesophageal reflux is unknown. Some studies suggest that both acid secretion and gastric motility are affected by emotions or stress, with some personality traits associated with labile basal acid secretion rate [13]. McDonald et al [14] also showed that relaxation training can alter esophageal acid exposure and reflux symptoms reports in patients with GERD.

Baker et al [15] did a psychological assessment in 51 patients with GERD and in 43 age-matched controls. His results suggested that although most patients with GERD were psychologically similar to patients without GERD, a subset of psychologically distressed patients (depression, somatization, anxiety and intensity of reporting symptom distress, n = 15) were more likely to be found among patients with GERD. Similarly, Johnson et al[16] reported a greater rate of phobia, obsessionality and somatization disorders in patients who seek medical attention for heartburn, than in those who do not consult. Kamolz et al[17] evaluated the outcomes of laparoscopic antireflux surgery in patients with known anxiety disorders. In that study, surgery improved the quality of life and corrected the acid reflux with however less symptoms relief in some of the patients.

These results suggest that psychological factors can influence the perception and/or the severity of GERD. In this specific study, there was no significant difference in the preoperative psychological assessment between patients suffering from GERD and the control group. However, a subset of patients experienced ongoing GERD symptoms and this specific group had a significantly higher level of somatization, as demonstrated by a higher SCL-90-R, somatization subset score.

### GERD and somatization disorders

Somatoform disorders are defined as psychiatric disorders in which one or more *unexplained *physical symptoms are the central defining feature of the disease. They frequently have abdominal manifestations, occurring in 17% to 28% of patients with irritable bowel syndrome[18]. They can also influence the surgical outcomes, by altering the patient's perception of pre- and post-operative symptoms and pain[19]. Clouse et al[20] demonstrated that patients with esophageal motility disorders score high on scales of somatic anxiety, depression and somatization. Baker et al[15] also found that somatization disorders are more likely to be found among patients with GERD.

In this specific study, the SCL-90-R, somatization subset was used to assess somatoform disorders. It is a self-report questionnaire, developed by Clinical Psychometric Research in 1976[21]. It is a reliable tool designed to reflect psychologic symptom patterns of individuals. The somatization subset used in this study measures somatization symptoms complex. Patients who experienced ongoing GERD symptoms after surgery had a significantly higher score on the somatization scale, compared to both the rest of the LNF Group and the Control Group.

### GERD and depression disorders

Depression is one of the most frequent psychological disorder in the general population. As a result, its impact on GERD and surgical outcomes has been evaluated before. Kamolz et al[22] compared GERD symptoms in patients who had both GERD and major depression to a control group, before and after laparoscopic fundoplication. Atypical GERD symptoms (chest pain: 81.6% vs 37.4% and bloating: 92.2% vs 37.4%) were more predominant and graded as much more severe among patients with major depression. Also, all symptoms but dysphagia significantly improved after laparoscopic antireflux surgery in both groups but patients with major depression had a significantly higher rate of chest pain, bloating, and dysphagia. Power et al[23], in a series of 131 patients who had a laparoscopic Nissen fundoplication, identified preoperative psychiatric history (major depression but also any psychogenic stressor) as an accurate predictor of LNF failure. Psychological history, especially with patients who have severe or refractory GERD symptoms, a positive review of systems or disrupted quality of life is thus essential. Patients with refractory GERD symptoms who have psychological issues and impaired quality of life may benefit more form integrated psychological care than referral to procedure oriented specialist[24].

In this specific study, there was no significant difference in the scores of Depression Anxiety Stress Scales and Beck Depression Inventory between any of the groups. However, five patients in the LNF group had abnormal Beck depression inventory's scores that could suggest depressive disorders. Three patients normalized their BDI score 6 months after surgery.

In conclusion, the SCL-90-R Somatization Subset, measuring the level of somatization in a patient, may be useful to predict poor outcomes after antireflux surgery. Cognizance of psychological disorders could improve the selection of an optimal treatment for GERD and help reduce the rate of ongoing symptoms after LNF. Larger studies are however required to confirm these results.
